# Early stress evokes age-dependent biphasic changes in hippocampal neurogenesis, epigenetic regulation of the bdnf gene, and cognitive behavior

**DOI:** 10.1186/1755-8166-7-S1-I38

**Published:** 2014-01-21

**Authors:** Vidita Vaidya

**Affiliations:** 1 Department of Biological Sciences, Tata Institute of Fundamental Research, Mumbai, India

## 

An experience of stress in early life is predominantly associated with negative consequences including increased anxiety and depressive behavior, as well as a failure to mount appropriate stress responses. It has remained a source of debate whether early stress also evokes potentially adaptive consequences that equip animals to cope better with their environment. We have shown that early stress exposure facilitates transient, adaptive changes in hippocampal neurogenesis, enhanced trophic factor expression and improved cognitive performance, thus providing possible competitive advantages in stressful environments. However, middle-aged animals with a history of early stress exhibit aladaptive effects on hippocampal neurogenesis, reduced trophic factor expression and impairments in cognitive performance. Our study provides novel insights into the short and long-term consequences of early stress, demonstrating biphasic, as well as unique, age-dependent changes at the molecular, epigenetic, neurogenic and behavioral level. These results compel a reappraisal of the traditional notion that early stress is deterministic for future negative outcomes. Our studies suggest that when observed across a life-span, early stress experience evokes both adaptive as well as maladaptive changes that emerge in a temporally regulated manner, with early adaptive outcomes that may eventually exert a high cost, evoking maladaptive consequences.

